# Statins for the Prevention of Stroke: A Meta-Analysis of Randomized Controlled Trials

**DOI:** 10.1371/journal.pone.0092388

**Published:** 2014-03-18

**Authors:** Wen Wang, Bo Zhang

**Affiliations:** Department of Neurosurgery, First Affiliated Hospital of Dalian Medical University, Dalian, PR China; University of Toronto, Canada

## Abstract

**Background:**

Stroke is a frequently encountered clinical event that has a detrimental impact on the quality of life. Evidence has increasingly shown that statins can substantially reduce the risk of coronary heart disease. However, it remains to be determined whether statins are definitively effective in preventing stroke.

**Methods:**

We systematically searched the PubMed, Embase, and Central databases for studies that compared the effects of statins and placebo in patients at high risk for stroke. The outcome measures were overall incidence of stroke, incidence of fatal stroke, and incidence of hemorrhagic stroke.

**Results:**

Eighteen randomized controlled trials satisfied all the inclusion criteria for the meta-analysis. The analysis revealed that statins reduced the overall incidence of stroke than placebo (odds ratio [OR]: 0.80; 95% confidence interval [CI]: 0.74–0.87; P<0.00001). In particular, statins showed efficacy in reducing the incidence of fatal stroke (OR: 0.90; 95% CI: 0.67–1.21; P = 0.47) and hemorrhagic stroke (OR: 0.87; 95% CI: 0.60–1.25; P = 0.45). On the contrary, they were found to increase the overall incidence of stroke (OR: 1.12; 95% CI: 0.89–1.41; P = 0.32) and fatal stroke (OR: 1.37; 95% CI: 0.93–2.03; P = 0.11) in renal transplant recipients and patients undergoing regular hemodialysis.

**Conclusion:**

The results of this analysis suggest that statins may be beneficial in reducing the overall incidence of stroke and they may decrease the risk of fatal stroke and hemorrhagic stroke. However, statins should be used with caution in patients with a history of renal transplantation, regular hemodialysis, transient ischemic attack, or stroke. Further analyses should focus on multicentre, double-blind, placebo-controlled randomized trials with data stratification according to the nature of primary diseases and dose–effect relationship, to clarify the benefits of statins in protection against stroke.

## Introduction

Stroke leads to disturbances in the blood supply to the brain, which can lead to the rapid deterioration of brain function. On the basis of etiology, stroke can be broadly classified into two types: ischemic and hemorrhagic; it is a heterogeneous condition that involves several causative factors in high-risk populations [1], such as patients with coronary heart disease (CHD), diabetes mellitus, and hypertension. A salient feature of stroke is that the type of stroke is not correlated with the prognosis of the patient [2].

Many studies have indicated that inhibitors of 3-hydroxy-3-methylglutaryl coenzyme A reductase (statins) reduce cardiovascular mortality by bringing about a reduction in the serum low-density lipoprotein (LDL) levels; this reduction has been shown to substantially lower the risk of CHD [3]–[6]. Further, several large-scale clinical trials have been conducted to evaluate the efficiency of statins in the primary and secondary prevention of atherosclerosis and stroke [7]–[9]. While some of these trials have shown the beneficial effect of statins in stroke prevention, others have not. This discrepancy in the currently available evidences leads to uncertainty regarding the effect of statins on the prevention of stroke in general and fatal stroke and hemorrhagic stroke in particular.

In the light of the prevalent confusion regarding the efficacy of statins in various high-risk populations, we sought to conduct a meta-analysis of randomized clinical trials (RCTs) evaluating the efficacy of statins in the primary and secondary prevention of stroke in high-risk populations.

## Methods

### Search strategy

In this meta-analysis, we conducted a thorough search of the PubMed, Embase, and Central databases for the reports of all the RCTs conducted up to October 2012 on the comparison of statins with placebo in the prevention of stroke, without any language restriction. The following search terms were used in various combinations: “stroke,” “pravastatin,” “lovastatin,” “atorvastatin,” “simvastatin,” “fluvastatin,” “cerivastatin,” “rosuvastatin,” “pitavastatin,” “HMG-CoA reductase inhibitor,” and “statins.” To account for both published and unpublished studies, we performed a cited references search by using Web of Science, checked the reference lists of the identified relevant trials, and contacted the authors of the respective papers and investigators. The primary endpoint of the analysis was the overall incidence of stroke, incidence of fatal stroke, and incidence of hemorrhagic stroke. The data extraction was independently performed by WW and BZ, and differences in opinion were resolved through discussion.

### Inclusion criteria

Studies were included in the meta-analysis if they fulfilled the following criteria: (1) enrolled subjects had high risk of stroke due to prevalent conditions (CHD, diabetes mellitus, hypertension, myocardial ischemia, and hypercholesterolemia) and were of mean age≥50 y; (2) the studies were RCTs conducted on humans; (3) the dosage of statin therapy was specified; (4) the details regarding the type of stroke, including fatal stroke and hemorrhagic stroke, were reported; and (5) the incidence of stroke in the study population was specified or could be calculated.

### Subgroup analysis

In order to specifically evaluate the efficacy of statins in patients with end-stage renal disease, a subgroup analysis was performed on trials that included only renal transplant recipients or patients undergoing regular hemodialysis.

### Quality assessment

The risk of bias in each study was evaluated by using the Cochrane Collaboration's tool following the instructions given in the Cochrane Handbook for Systematic Reviews. The assessment was made across six domains: sequence generation, allocation concealment, blinding, incomplete data outcomes, selective outcome reporting, and other causes of bias. We studied the influence of the methodological quality of the trials on their results by reviewing the reported randomization protocol and follow-up procedures adopted in each trial.

The quality of evidence was rated using the Grade of Recommendation, Assessment, Development, and Evaluation (GRADE) approach by using the GRADEpro software (version 3.6). As per the GRADE approach, the evidences were graded into the following levels of quality according to the likelihood of change in the estimate of the effect in the light of further research: (1) high quality, if the estimate was extremely unlikely to change; (2) moderate quality, if the estimate was moderately likely to change; (3) low quality, if the estimate effect was highly likely to change; (4) very low quality, if the estimate appeared to be extremely uncertain.

### Statistical analysis

The overall incidence of stroke was expressed in dichotomous variables, and the results were expressed as odds ratio (OR) with 95% confidence interval (CI). Data on the incidence of fatal stroke and hemorrhagic stroke were available in 12 and 11 trials, respectively. The pooled estimate of efficacy was calculated using the Mantel-Haenszel method, and the random-effects model was used because various types and strengths of statins were used in the analyzed studies. Significant heterogeneity was defined at P<0.05. Heterogeneity was assessed using the I^2^ statistic: when I^2^ was<25%, heterogeneity was considered absent; when I^2^ was 25–50%, heterogeneity was considered low; when I^2^ was 50–75%, heterogeneity was considered moderate; and when I^2^ was>75%, heterogeneity was considered high. All statistical analyses were performed using Review Manager 5.2 (version 5.2.4; http://ims.cochrane.org/revman).

## Results

### Literature search

**Figure 1:**
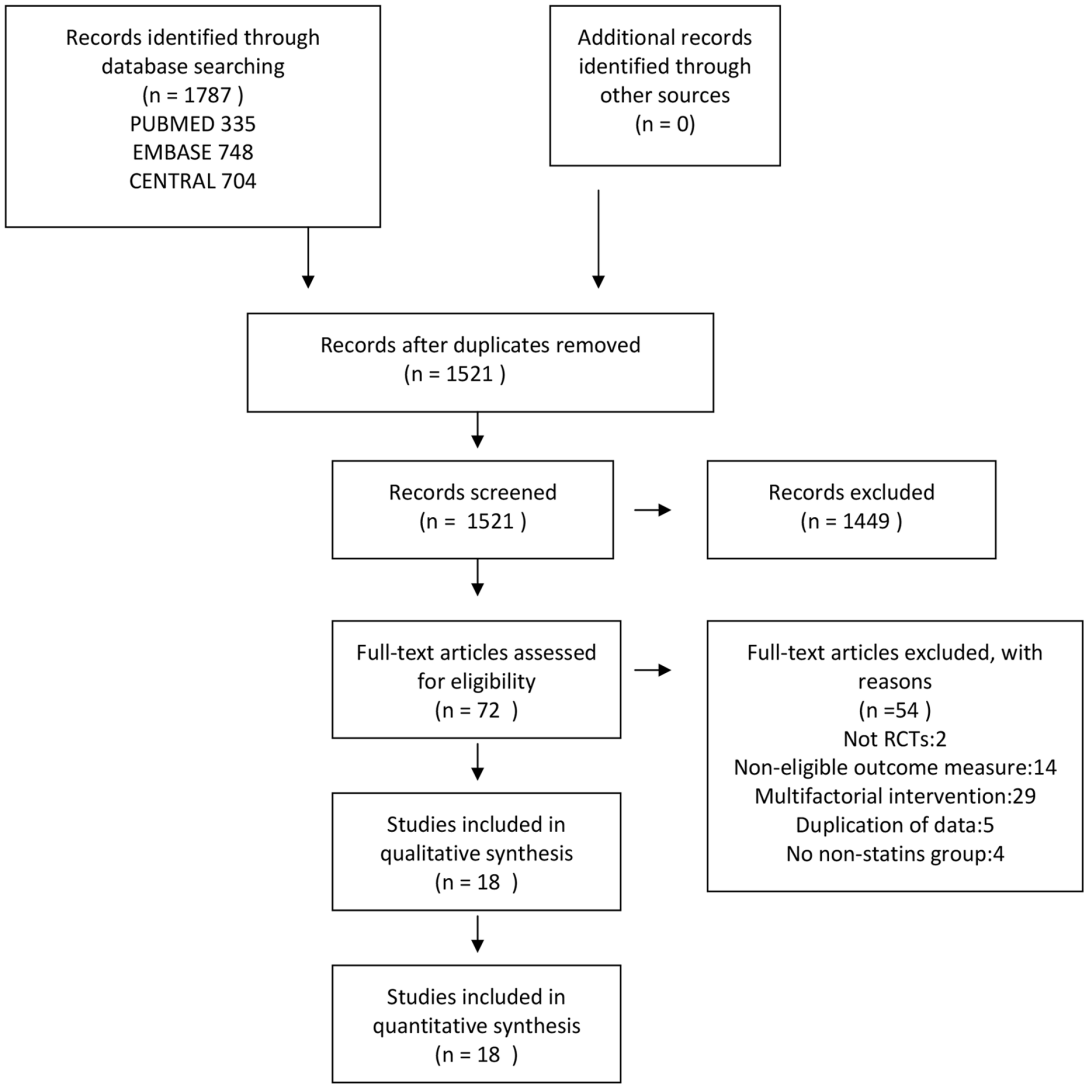
Flow chart indicating the selection process for this meta-analysis.

The initial database search retrieved 1787 studies (335 from PubMed, 748 from Embase, 704 from Central) that were limited to humans, RCTs, and published before October 2012. After eliminating duplicate entries, the number of entries was reduced to 1521. Finally, after applying all the inclusion criteria, 18 RCTs, conducted on 114,081 subjects in all, were selected for the analysis (Figure 1).

### Study characteristics

The salient features of the 18 selected studies [10]–[27] are summarized in Table 1. The studies were published between 1999 and 2010, included 1255 to 20536 subjects each, and had a mean follow-up duration of 4 y. Various statins were investigated in these trials: rosuvastatin, fluvastatin, atorvastatin, pravastatin, and simvastatin. The mean serum level of LDL recorded in the studies was 136 mg/dl.

**Table 1:** Baseline characteristics of included studies.

STUDY ID	AGE	FEMALE %	TREATMENT	Follow-up year	N(I/C)	Overall Stroke(OS)	Fatal Stroke(FS)	Hemorrhagic Stroke(HS)
Athyros VG et al. 2002 [10]	59 y	22%	Atorvastatin 10–80	3 y	800/800	9/17	ND	ND
Koren MJ et al. 2004 [11]	61 y	17%	Simvastatin 10–80	4.3 y	1217/1225	35/39	ND	ND
Knopp RH et al. 2006 [12]	61 y	34%	Simvastatin 10	4 y	1211/1199	34/38	ND	ND
Sever PS et al. 2007 [13]	63 y	19%	Atorvastatin 10	3.3 y	5168/5163	110/139	ND	ND
White HD et al. 2000 [14]	62 y	17%	Pravastatin 40	6 y	4512/4502	169/204	ND	9/18
Nakamura H et al. 2006 [15]	58 y	69%	Simvastatin 10–20	5.3 y	3866/3966	50/62	ND	16/14
ALLHAT 2002 [16]	66 y	49%	Pravastatin 40	4.8 y	5170/5185	209/231	53/56	ND
Shepherd J et al. 2002 [17]	75 y	52%	Pravastatin 40	3.2 y	2891/2913	135/131	22/14	ND
Hitman GA et al. 2007 [18]	62 y	32%	Atorvastatin 10	3.9 y	1428/1410	21/39	1/7	ND
Amarenco P et al. 2006 [19]	63 y	40%	Simvastatin 80	4.9 y	2365/2366	265/311	24/41	55/33
Plehn JF et al. 1999 [20]	59 y	14%	Pravastatin 40	5 y	2081/2078	52/76	5/1	2/6
HPSI 2003 [21]	65 y	25%	Simvastatin 40	5 y	10269/10267	444/585	96/119	51/53
Waters DD et al. 2002 [22]	65 y	35%	Atorvastatin 80	0.3 y	1538/1548	13/25	3/2	0/3
Kjekshus J et al. 2007 [23]	73 y	24%	Simvastatin 10	2.7 y	2514/2497	103/115	14/11	15/9
Everett BM et al. 2010 [24]	66 y	38%	Rosuvastatin 20	1.9 y	8901/8901	33/64	3/6	6/9
Wanner C et al. 2005 [25]	66 y	46%	Simvastatin 20	4 y	619/636	60/45	27/13	3/5
Fellstrom BC et al. 2009 [26]	64 y	38%	Simvastatin 10	3.2 y	1389/1384	93/81	40/36	25/21
Abedini S et al. 2009 [27]	50 y	34%	Fluvastatin 80	6.7 y	1050/1052	77/83	21/17	10/17

ND: No Data; I/C: Intervention/Control.

All the studies were conducted on populations at high risk of stroke, including those with CHD, diabetes mellitus, hypertension and myocardial ischemia. Additionally, one RCT [27] included renal transplant recipients, and two RCTs [25], [26] included patients undergoing regular hemodialysis. A subgroup analysis was conducted with these three RCTs. In addition, patients in one of the RCTs [19] had history of stroke or transient ischemic attack (TIA) within the past one to six months, and statins were used in this population for the secondary prevention of stroke.

### GRADE evidence profile

All the included RCTs had the same endpoints, which were overall incidence of stroke, incidence of fatal stroke, and incidence of hemorrhagic stroke. The GRADE evidence profiles for upgrading or downgrading each outcome level are shown in Table 2.

**Table 2:** GRADE profile evidence of the included studies.

Quality assessment							No of patients		Effect		Quality	Importance
No of studies	Design	Risk of bias	Inconsistency	Indirectness	Imprecision	Other considerations	Statins	Control	Relative (95% CI)	Absolute		
**Overall Stroke (follow-up mean 4 years)**												
18	Randomized trials	No serious risk of bias	No serious inconsistency	No serious indirectness	No serious imprecision	Reporting bias	1912/56989 (3.4%)	2285/57092 (4%); 4.1%	OR 0.84 (0.76 to 0.92)	6 fewer per 1000 (from 3 fewer to 9 fewer); 6 fewer per 1000 (from 3 fewer to 10 fewer)	MODERATE	IMPORTANT
**Fatal Stroke (follow-up mean 3.8 years)**												
12	Randomized trials	No serious risk of bias	No serious inconsistency	No serious indirectness	No serious imprecision	Reporting bias	309/40215 (0.8%)	323/40237 (0.8%); 0.8%	OR 1.03 (0.79 to 1.35)	0 more per 1000 (from 2 fewer to 3 more); 0 more per 1000 (from 2 fewer to 3 more)	MODERATE	CRITICAL
**Hemorrhagic Stroke (follow-up mean 4 years)**												
10	Randomized trials	No serious risk of bias	No serious inconsistency	No serious indirectness	No serious imprecision	Reporting bias	137/36739 (0.4%)	155/36831 (0.4%); 0.4%	OR 0.88 (0.67 to 1.15)	1 fewer per 1000 (from 1 fewer to 1 more); 0 fewer per 1000 (from 1 fewer to 1 more)	MODERATE	CRITICAL

### Risk of bias

**Figure 2:**
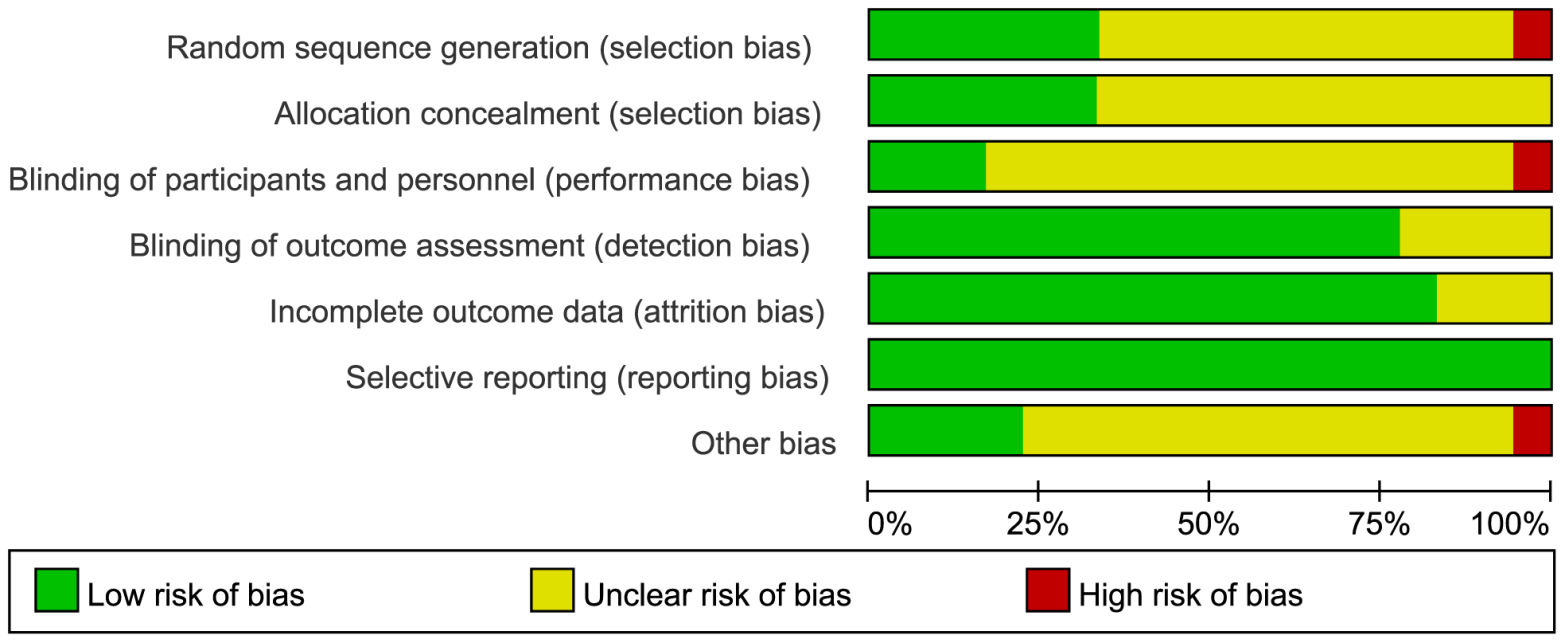
Risk of bias graph.

**Figure 3:**
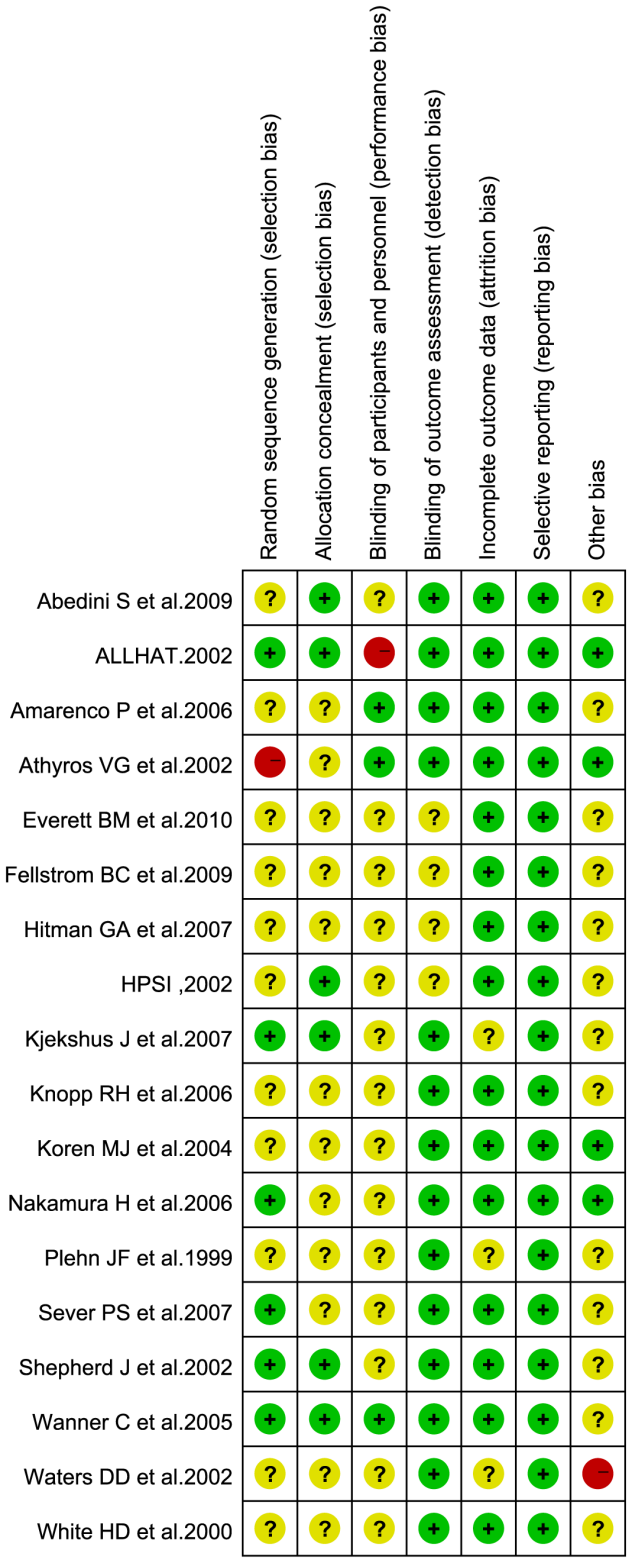
Risk of bias summary.

The risk of bias in the included studies is summarized through a graph (Figure 2) and summary (Figure 3). The 18 trials were conducted across eight different countries, namely, USA, UK, Norway, Sweden, Greece, Japan, Germany, and New Zealand, and were mostly based in hospitals or clinics. Only four of the included trials [16], [17], [23], [25] had adequate allocation concealment. All the included trials were considered to have adequate sequence generation because they were essentially randomized in nature. The reports of 12 trials did not describe the method used for generating the allocation sequence. One trial [10] report indicated that patients were randomly allocated to the intervention or placebo group at the out-patient clinic, and therefore, the risk of bias for random sequence generation was considered to be high. While most trials reported blinding of outcome assessment, that of participants, personnel, and outcome assessment was reported only in the case of three [10], [19], [25] trials. One study [16] was a nonblinded trial, and the risk of bias due to inadequate blinding of participants and personnel was considered high in this case. In most of the selected RCTs, participant flow and important outcomes were reported; therefore, we assessed the incomplete outcome data bias and considered all trials to be of low risk of selective reporting. In one trial [22], the duration of follow up was only 0.3 y, which made the long-term effect of statins on stroke incidence unclear; therefore, the risk of bias due to other causes was considered to be high for that trial.

### Overall stroke incidence

**Figure 4:**
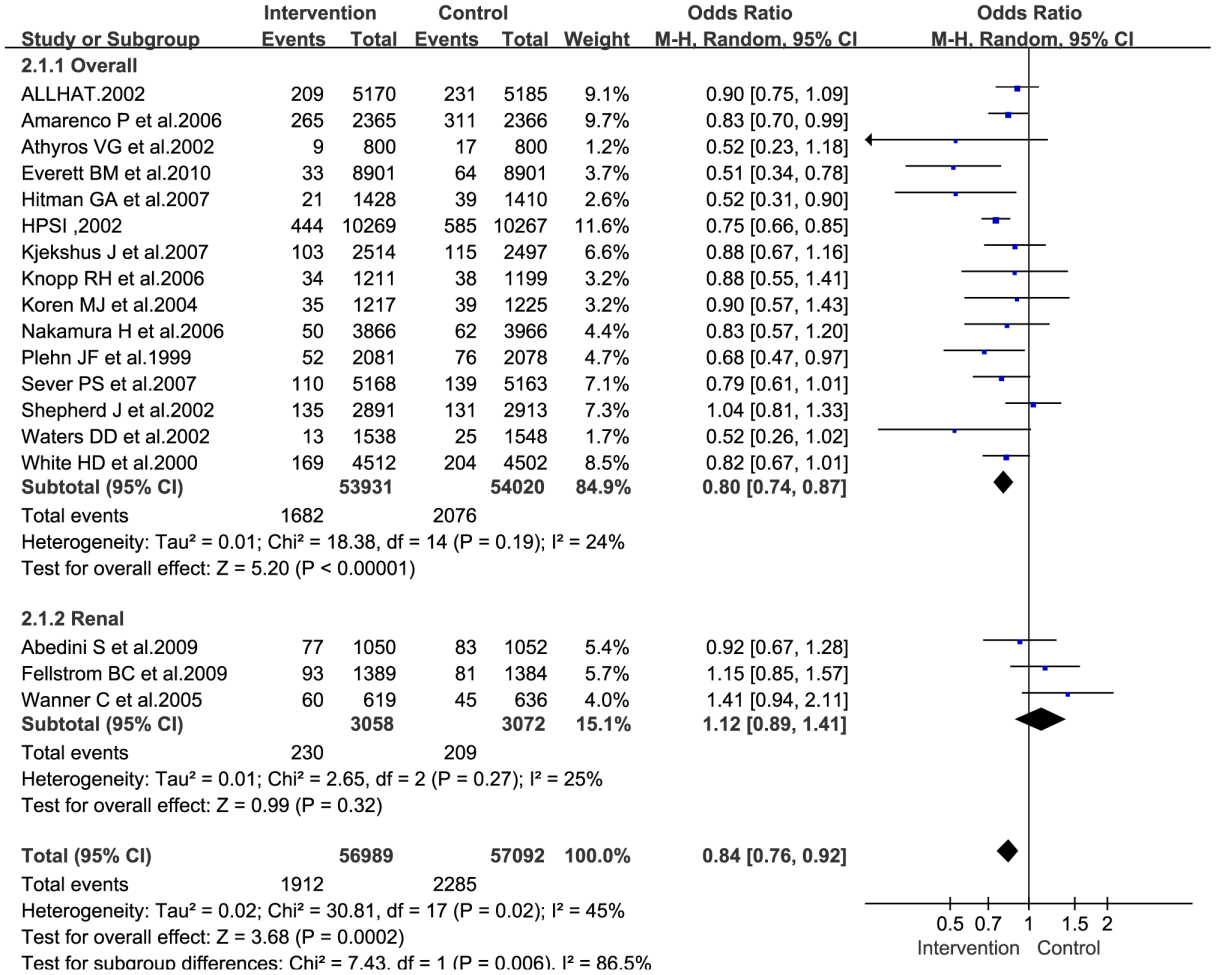
Forest plot for overall stroke incidence.

The overall incidence of stroke was indicated in all the trial reports. The pooled percentages in the intervention and placebo groups were 3.36% and 4%, respectively. The result of the meta-analysis of all the included studies showed a significant reduction in the incidence of overall stroke in patients treated with statins (OR: 0.80; 95% CI: 0.74–0.87; P<0.00001; Figure 4).

The trial by Amarenco et al. [19] included patients with history of stroke or TIA. Pooled results obtained after excluding this study did not differ significantly from those obtained after its inclusion (OR: 0.80; 95% CI: 0.72–0.87; P<0.00001), and neither did the heterogeneity (I^2^ = 28%, heterogeneity, P = 0.15).

### Fatal stroke incidence

**Figure 5:**
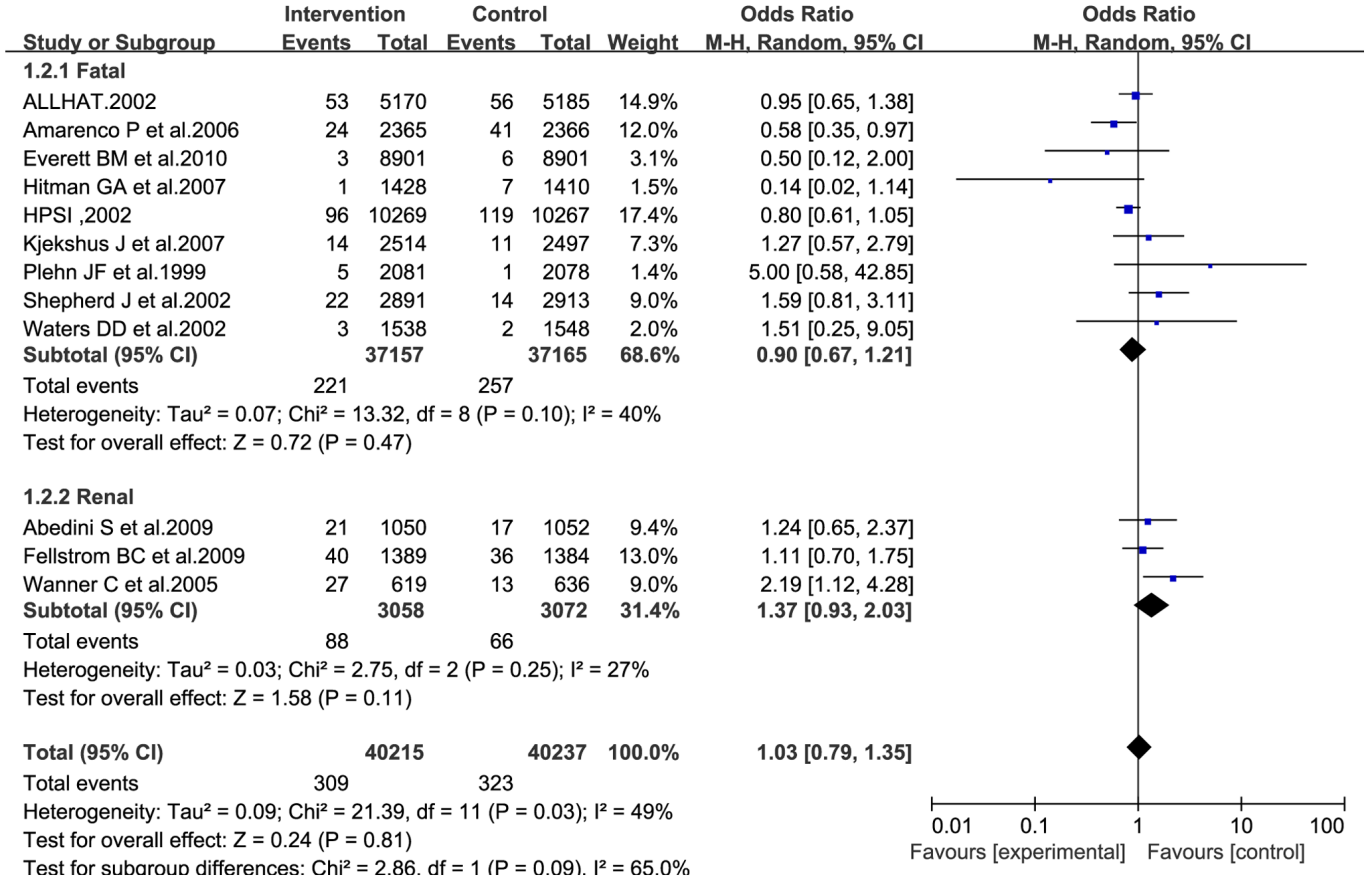
Forest plot for fatal stroke incidence.

Nine trial reports provided data on the incidence of fatal stroke [16]–[24] among 74,322 patients (Table 1). Meta-analysis using the random-effects model showed that statin treatment induced no significant reduction in the incidence of fatal stroke (OR: 0.90; 95% CI: 0.67–1.21; P = 0.47; Figure 5) and that heterogeneity among the trials was low (I^2^ = 40%; heterogeneity, P = 0.10).

### Hemorrhagic stroke incidence

**Figure 6:**
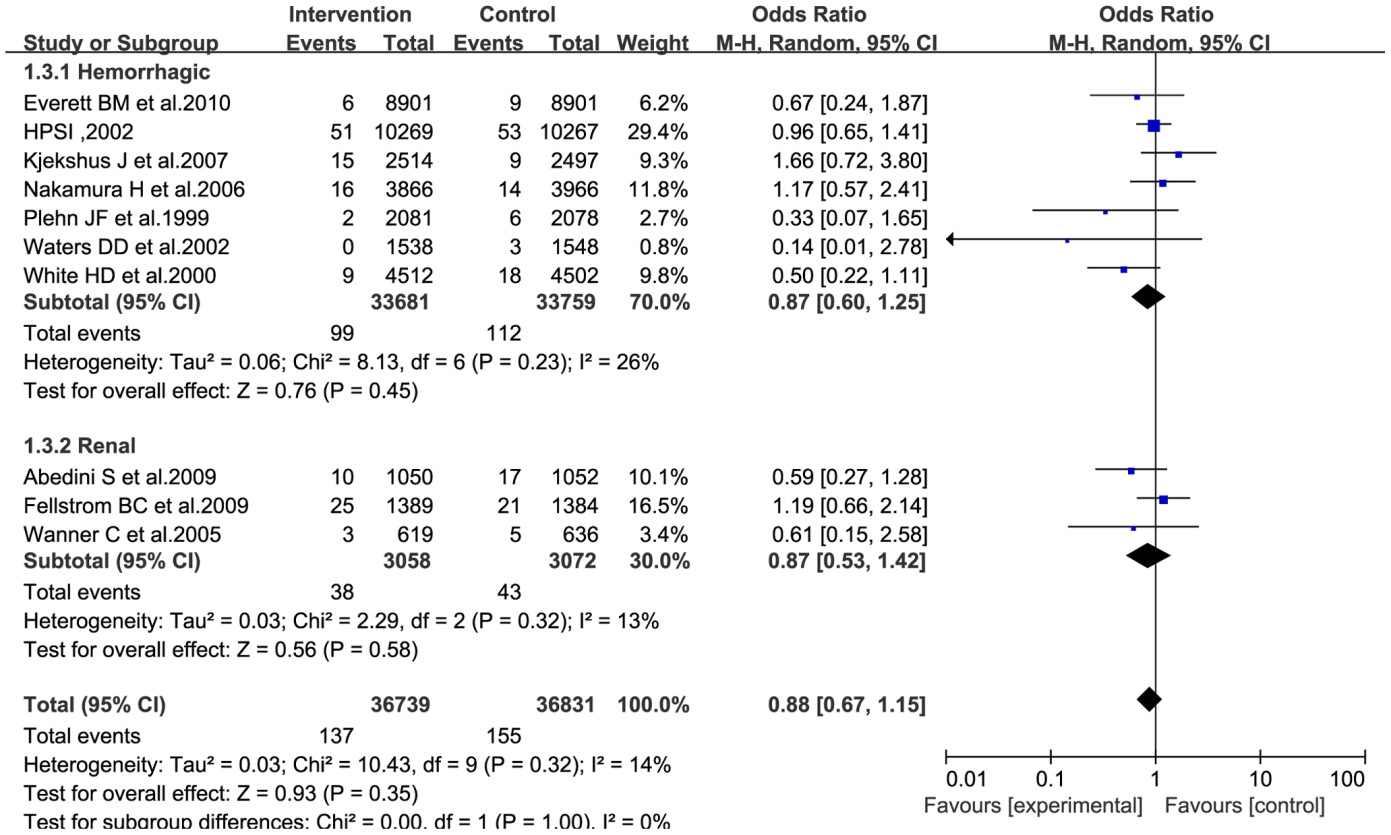
Forest plot for hemorrhagic stroke incidence.

Data regarding the efficiency of statins in the prevention of hemorrhagic stroke were available for 11 RCTs. Pooled analysis of data from 7 [14], [15], [20]–[24] of these studies, using the random-effects model, revealed that statins did not significantly reduce the incidence of hemorrhagic stroke (OR: 0.87; 95% CI: 0.60–1.25; P = 0.45; Figure 6) and had low statistical heterogeneity (I^2^ = 26%; heterogeneity, P = 0.23). Analysis including the study performed by Amarenco et al. [19] showed a different result (OR: 0.98; 95% CI: 0.67–1.43; P = 0.91), with low heterogeneity (I^2^ = 49%; heterogeneity, P = 0.06).

### Subgroup analysis

Subgroup analysis was performed on the three trials [25]–[27] conducted on renal transplant recipients or patients undergoing regular hemodialysis, by using the random-effects model. The analysis revealed the following: statins reduced the overall incidence of stroke, although this reduction had low statistical significance (OR: 1.12; 95% CI: 0.89–1.41; P = 0.32; Figure 4); statins may, in fact, increase the incidence of fatal stroke (OR: 1.37; 95% CI: 0.93–2.03; P = 0.11; Figure 5); and statins had a beneficial effect on the incidence of hemorrhagic stroke (OR: 0.87; 95% CI: 0.53–1.42; P = 0.58; Figure 6), but with low statistical significance.

### Publication bias

**Figure 7:**
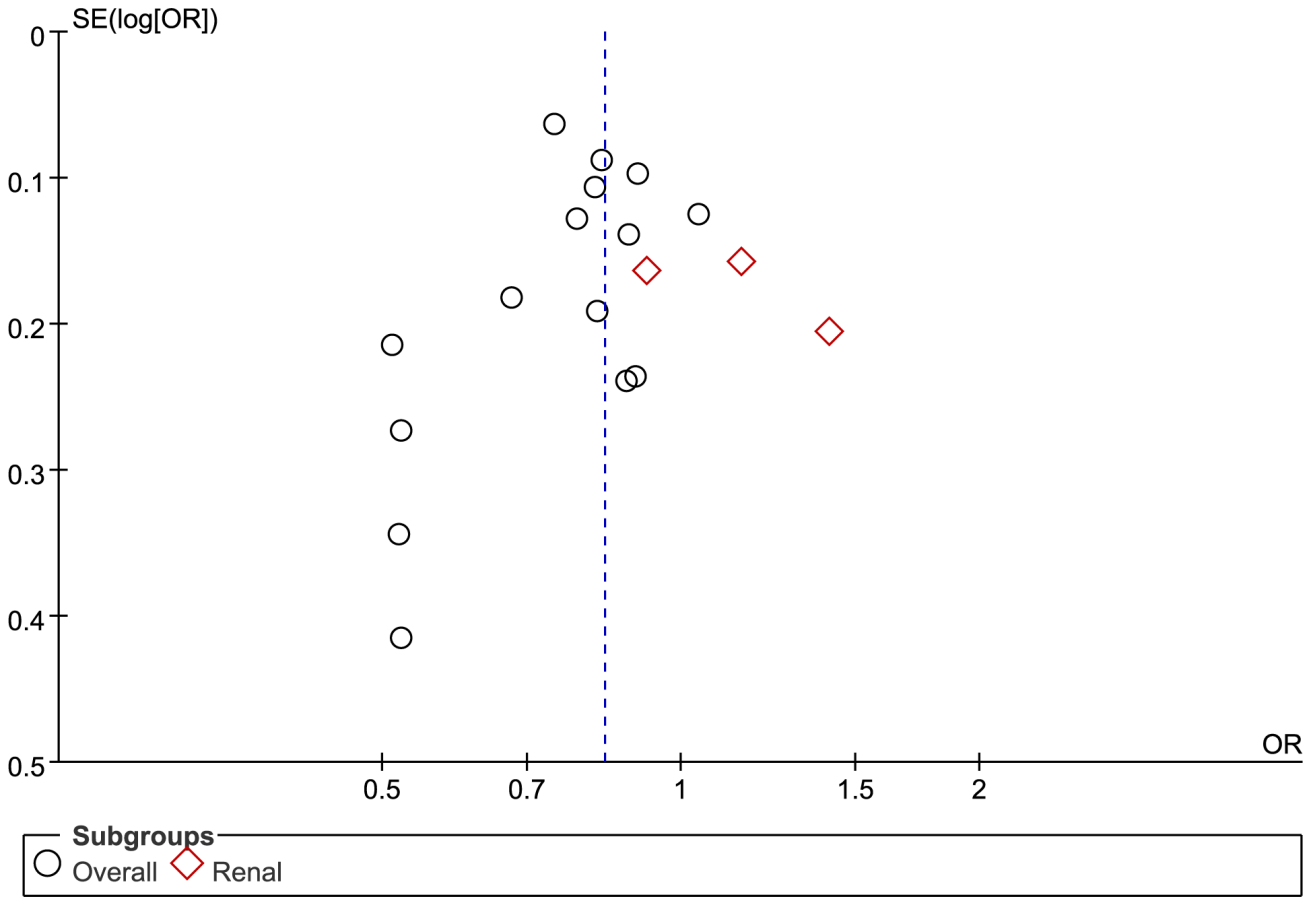
Funnel plot for 18 randomized controlled trials.

Publication bias was assessed using the funnel plot, and the results indicated that the risk of significant bias was low (Figure 7).

## Discussion

Current evidences indicate that statins can reduce the incidence of cardiovascular disease via various mechanisms, which include reduced lipid and platelet aggregation, improved endothelial function, anti-inflammation activity, and neuroprotective action [28]–[31]. Through this meta-analysis, we sought to determine whether statins can indeed prevent stroke, especially fatal stroke and hemorrhagic stroke.

Our analysis of 18 RCTs revealed that statins did, in fact, significantly reduce the overall incidence of stroke. Further, statins were found to be effective in the prevention of fatal stroke, although this effect was not statistically significant. On the contrary, three RCTs [25]–[27] showed that statins may potentially increase the incidence of overall stroke and fatal stroke in patients with a history of renal transplantation, regular hemodialysis, TIA, or stroke, but not significantly. Since this finding was not statistically significant, further investigations are warranted to confirm this. Nevertheless, statins have been previously shown to protect against kidney disease through various immunomodulatory mechanisms [7]–[9], [32]. Therefore, when treating kidney transplant recipients or patients undergoing regular hemodialysis, clinicians should carefully assess the requirement and the dosage of statins administered in relation to the patient's renal function.

This meta-analysis revealed that the use of statins may decrease the incidence of hemorrhagic stroke, although not in a statistically significant manner. This finding is consistent with previous reports indicating the safety of statins in the prevention of hemorrhagic stroke [33], [34]. A study by Amarenco et al. [19] on the secondary prevention of stroke revealed that patients treated with statins had a significantly greater frequency of hemorrhagic stroke (55 events) than those treated with placebo (33 events), thereby indicating that the incidence of hemorrhagic stroke in the intervention group was 67% (95% CI: 1.09–2.60). Subsequent analysis revealed that the incidence of hemorrhagic stroke was particularly high in older male patients who had a history of hypertension or stroke [35]. This may be explained by the fact that statins are reported to cause vascular dilatation with rising levels of nitric oxide in the vascular endothelium, which has been implicated in the pathogenesis of hemorrhagic stroke. This may render statins unsuitable for the secondary prevention of stroke.

With regard to the quality of evidence, evaluation using the GRADE system indicated that the data from the included studies were of moderate quality. Since all the 18 included RCTs yielded important outcomes, all trials were to be at low risk of selective reporting.

Our findings should be interpreted in the light of a few limitations. This meta-analysis included only three RCTs comprising renal transplant recipients or patients undergoing regular hemodialysis and only one trial comprising patients with a history of TIA or stroke. This could have led to an underestimation or overestimation of the true incidence of stroke among the analyzed population. Further, we could not account for the impact of the type of coexisting primary diseases (e.g., CHD, diabetes mellitus, hypertension etc.), because the evaluated reports did not contain separate records for the various conditions. Another drawback is that from the current evidences, we were unable to analyze the possible dose–effect relationship for different types of statins. This highlights the need for more multicentre, double-blind, placebo-controlled randomized trials focusing on the nature of the coexisting primary disease and dose–effect relationship.

## Conclusion

The findings of this meta-analysis indicate that statins may be beneficial in preventing the occurrence of stroke in general. In particular, it may potentially reduce the incidence of fatal stroke and hemorrhagic stroke. However, caution must be exercised when using statins in patients with a history of renal transplantation, regular hemodialysis, TIA, or stroke. Further analyses based on data collected in multicentre, double-blind, placebo-controlled, randomized trials and stratified by primary diseases and dose–effect relationship are warranted to substantiate the findings of this meta-analysis.

## Supporting Information

Protocol S1 (DOC)

Checklist S1 (DOC)

Search Strategy S1 (DOC)
