# Supplementary material for: Statins for the Prevention of Stroke: A Meta-Analysis of Randomized Controlled Trials
Source: PLoS One. 2014 Mar 18;9(3):e92388. doi: 10.1371/journal.pone.0092388 (PMC3958535; doi:10.1371/journal.pone.0092388)
Supplement: Protocol S1 — (DOC) [file pone.0092388.s001.doc]

**Statins for the prevention of stroke: a meta-analysis of randomized controlled trials**

**Wen Wang^1^, Bo Zhang^1^***

1.Department of Neurosurgery, First Affiliated Hospital of Dalian Medical University, Dalian, PR China

* E-mail: [zhangbodl@126.com](mailto:zhangbodl@126.com)

**Background:**

Stroke is a common event associated with a detrimental impact on the quality of lives. An increasing body of evidence demonstrates that the statins can reduce the risk for coronary heart disease substantially. It is uncertain whether statins could effectively prevent stroke, especially fatal stroke and hemorrhagic stroke.

The inhibitors of 3-hydroxy-3-methylglutaryl coenzyme A ( HMG-CoA ) reductase ( statins ) were widespreadly accepted as the primary and secondary prevention of atherosclerosis and stroke. Statins can reduce serum low-density lipoprotein ( LDL ) levels, so it could decrease the cardiovascular mortality.

It is unclear about the effect of statins for the prevention of overall stroke, especially for the fatal stroke and hemorrhagic stroke. So we made a meta-analysis focusing on overall stroke, fatal stroke and hemorrhagic stroke to assess its effect.

**Objectives:**

The aim of the study was to assess the effect of statins for the prevention of stroke in targeted randomized controlled trials (RCTs) with patients who got high risks of strokes.

**Methods:**

**Type of studies**

Only randomized controlled trials were included in our meta-analysis. The RCTs in which the incidence was reported or can be calculated were eligible for the inclusion of the analysis.

**Types of participants**

The included participants all have high risks of strokes. The patients included in the analysis have different primary diseases, such as coronary heart disease, diabetes mellitus, hypertension, myocardial ischemia and hypercholesterolemia. We would analyze the trials in which the patients got renal transplantations or regular hemodialysis in subgroup. Mean age of the participants≥50.

**Types of interventions**

There are a number of statins on the market now, including: pravastatin, lovastatin, atorvastatin, simvastatin, fluvastatin, cerivastatin, rosuvastatin and pitavastatin. No restricted to types of statin.

**Types of outcome measures**

The outcome of the analysis were statins for the prevention of overall stroke(OS), fatal stroke(FS) and hemorrhagic stroke(HS). The data of strokes was extracted or calculated from targeted RCTs.

**Databases and search strategy**

Databases included PUBMED and EMBASE. Results were limited to human, randomized controlled trials and there were no restricted to language.

The CENTRAL database should also been searched.

In order to identify further published and unpublished studies, we carried out a Cited Reference Search using Web of Science, checked the reference lists of identified relevant trials and contacted authors of relevant papers and investigators in this area of stroke services trials.

Search strategy: Search terms included: "stroke", "pravastatin", "lovastatin", "atorvastatin", "simvastatin", "fluvastatin", "cerivastatin", "rosuvastatin", "pitavastatin", "HMG-CoA reductase inhibitor", "statins".

*Example: PUBMED*

("Stroke"[Mesh] OR stroke or strokes or “hemorrhagic stroke” or “haemorrhagic stroke” or “ischemic stroke” or “ischaemic stroke” or “fatal stroke” or "brain ischemia" or "brain hemorrhage" or "brain ischaemia" or "brain haemorrhage" or "cerebrovascular accident") AND ("Hydroxymethylglutaryl-CoA Reductase Inhibitors"[Mesh] OR “HMG-CoA reductase inhibitor” OR hydroxymethylglutaryl* OR HMG-CoA* OR statin OR statins OR pravastatin OR lovastatin OR atorvastatin OR simvastatin OR fluvastatin OR cerivastatin OR rosuvastatin OR pitavastatin) AND ((randomized controlled trial[pt] OR controlled clinical trial[pt] OR randomized[tiab] OR placebo[tiab] OR “clinical trials as topic”[Mesh: noexp] OR randomly[tiab] OR trial[ti]) NOT (animals[mh] NOT (humans[mh] AND animals[mh])))

publication data to 2012/10/31, human, randomized controlled trails

**Selection of studies**

Details in Flow Chart.


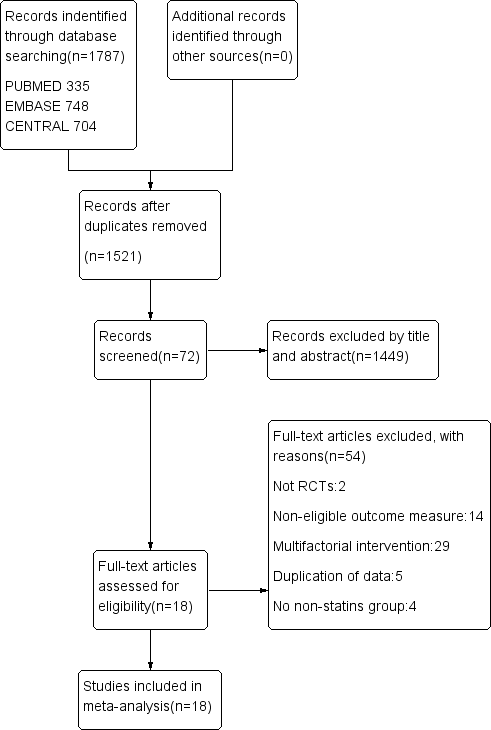


Two investigators(WW, BZ) searched the PUBMED, EMBASE and CENTRAL databases independently to identify studies featuring statins, stroke, fatal stroke and hemorrhagic stroke, which published before October 2012. We excluded papers that did not meet the inclusion criteria at final stage and recorded the reason.

If there were any differences in opinion about studies inclusion, we resolved them through discussion.

**Data extraction and management**

Two authors(WW, BZ) independently identified the trials for inclusion and extracted the data. We calculated the odd ratio ( OR ) with 95% confidence intervals using the fixed-effect and the random-effects models based on heterogeneity using RevMan 5. The meta-analysis was performed with the Random-effects model because of the variety of statin medication and the variability in the dosing of the medications.

WW and BZ, independently of each other, extracted the following data:

1. Year and language of publication.

2. Country.

3. Follow-up of trial.

4. Inclusion and exclusion criteria.

5. Sample size.

6. Population characteristics such as age and sex ratio.

7. Types and dose of statins.

8. Outcomes (Overall stroke, Fatal stroke and Hemorrhagic stroke).

9. Methodological quality (described below).

Any differences in data extraction and management were resolved through discussion.

**Assessment of methodological quality**

The authors assessed the methodological quality of the trials independently without masking of the trial names and followed the instructions given in the Cochrane Handbook for Systematic Reviews (Section 8.5). We looked at the influence of methodological quality of the trials on the results by evaluating the reported randomization and follow-up procedures in each trial. We assessed sequence generation, allocation concealment, blinding, incomplete data outcomes, selective outcome reporting, and other biases.

**Outcome measures**

The outcome measures reported by the included trials was incidence of overall stroke, fatal stroke and hemorrhagic stroke. The outcome measures reported by the individual trials are shown in the Table 1 (Baseline characteristics of included studies).

**Unit of analysis issues**

1 Overall stroke (Figure 4)


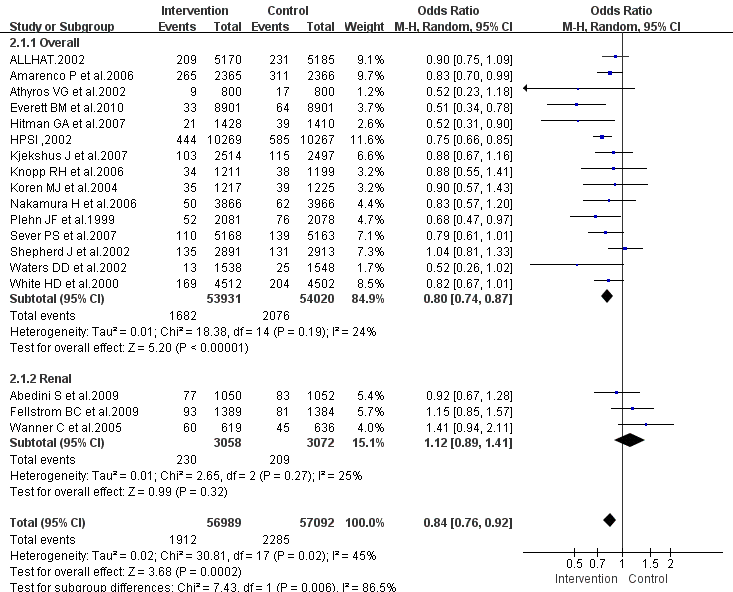


2 Fatal stroke (Figure 5)


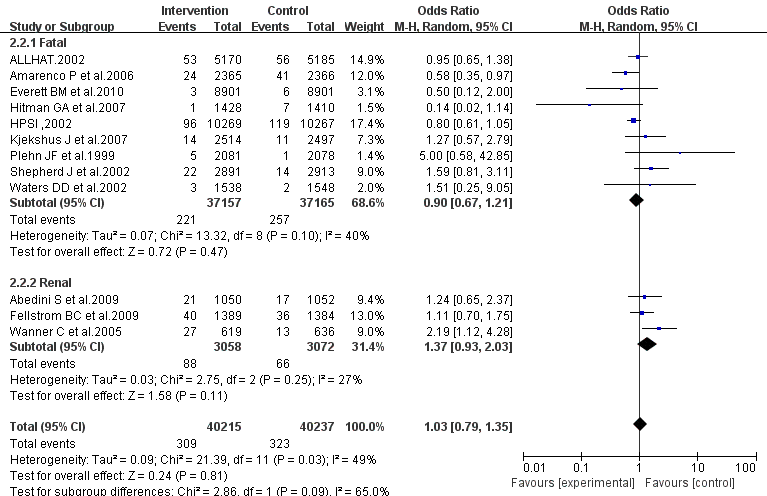


3 Hemorrhagic stroke (Figure 6)


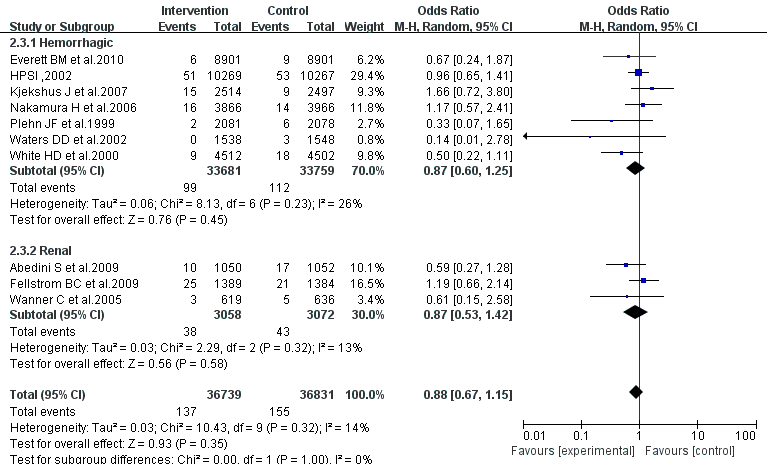


**Effects of interventions**

The results are reported as odd risk (OR) with 95%confidence intervals (CI).

**Subgroup analysis**

We analyze the trials in which the patients got renal transplantations or regular hemodialysis in subgroup.

**The quality of the evidence**

Use GRADE to assess the quality of the evidence according to the Cochrane Handbook of Systematic reviews(Chapter 12) by using GRADEprofiler. There are five reasons for downgrading the quality of a body of evidence, including Limitations in the design and implementation, Indirectness of evidence, inconsistency of results, Imprecision of results, publication bias.

**Author Contributions**

WW and BZ performed the literature search and the data extraction independently. WW wrote the paper and was responsible for the final approval of the version to be published. Conceived and designed the experiments by BZ and WW.

**Funding**

The authors have no support or funding to report.
