# Supplementary material for: Statins for the Prevention of Stroke: A Meta-Analysis of Randomized Controlled Trials
Source: PLoS One. 2014 Mar 18;9(3):e92388. doi: 10.1371/journal.pone.0092388 (PMC3958535; doi:10.1371/journal.pone.0092388)
Supplement: Search Strategy S1 — (DOC) [file pone.0092388.s003.doc]

PUBMED

("Stroke"[Mesh] OR stroke or strokes or “hemorrhagic stroke” or “haemorrhagic stroke” or “ischemic stroke” or “ischaemic stroke” or “fatal stroke” or "brain ischemia" or "brain hemorrhage" or "brain ischaemia" or "brain haemorrhage" or "cerebrovascular accident") AND ("Hydroxymethylglutaryl-CoA Reductase Inhibitors"[Mesh] OR “HMG-CoA reductase inhibitor” OR hydroxymethylglutaryl* OR HMG-CoA* OR statin OR statins OR pravastatin OR lovastatin OR atorvastatin OR simvastatin OR fluvastatin OR cerivastatin OR rosuvastatin OR pitavastatin) AND ((randomized controlled trial[pt] OR controlled clinical trial[pt] OR randomized[tiab] OR placebo[tiab] OR “clinical trials as topic”[Mesh: noexp] OR randomly[tiab] OR trial[ti]) NOT (animals[mh] NOT (humans[mh] AND animals[mh])))

publication data to 2012/10/31, human, randomized controlled trails

CENTRAL

#1 MeSH descriptor:[Stroke] explode all trees

#2 stroke or strokes or “hemorrhagic stroke” or “haemorrhagic stroke” or “ischemic stroke” or “ischaemic stroke” or “fatal stroke” or "brain ischemia" or "brain hemorrhage" or "brain ischaemia" or "brain haemorrhage" or "cerebrovascular accident"

#3 #1 or #2

#4 MeSH descriptor: [Hydroxymethylglutaryl-CoA Reductase Inhibitors] explode all trees

#5 hydroxymethylglutaryl*

#6 HMG-CoA*

#7 statin or statins

#8 pravastatin or lovastatin or atorvastatin or simvastatin or fluvastatin or cerivastatin or rosuvastatin or pitavastatin

#9 (#4 or #5 or #6 or #7 or #8)

#10 #3 and #9 to 2012

EMBASE

#21 #20 AND "randomized controlled trial"/de AND "human"/de

#20 #3 AND #9 AND #18 NOT [31-10-2012]/sd

#19 #3 AND #9 AND #18

#18 #10 OR #11 OR #12 OR #13 OR #14 OR #15 OR #16 OR #17

#17 "randomized controlled trial":ab,ti

#16 trial:ab,ti

#15 randomly:ab,ti

#14 placebo:ab,ti

#13 randomized:ab,ti

#12 "randomized controlled trial"/exp

#11 "randomized controlled trial (topic)"/exp

#10 random*

#9 #4 OR #5 OR #6 OR #7 OR #8

#8 "pravastatin"/exp OR "lovastatin"/exp OR "atorvastatin"/exp OR "simvastatin"/exp OR "fluvastatin"/exp OR "cerivastatin"/exp OR "rosuvastatin"/exp OR "pitavastatin"/exp

#7 "statin"/exp OR "statins"/exp

#6 "hmg coa":ab,ti

#5 hydroxymethylglutaryl*

#4 "hydroxymethylglutaryl coenzyme a reductase inhibitor"/exp

#3 #1 OR #2

#2 stroke:ab,ti OR strokes:ab,ti OR "hemorrhagic stroke":ab,ti OR "haemorrhagic stroke":ab,ti OR "ischemic stroke":ab,ti OR "ischaemic stroke":ab,ti OR "fatal stroke":ab,ti OR "brain ischemia":ab,ti OR "brain hemorrhage":ab,ti OR "brain ischaemia":ab,ti OR "brain haemorrhage":ab,ti OR "cerebrovascular accident":ab,ti

#1 "cerebrovascular accident"/exp
